# Integrated control of bacterial growth and stress response by (p)ppGpp in *Escherichia coli*: A seesaw fashion

**DOI:** 10.1016/j.isci.2024.108818

**Published:** 2024-01-09

**Authors:** Manlu Zhu, Haoyan Mu, Xiongfeng Dai

**Affiliations:** 1 Hubei Key Laboratory of Genetic Regulation and Integrative Biology, School of Life Sciences & National Key Laboratory of Green Pesticides, Central China Normal University, Wuhan, China

**Keywords:** Molecular biology, Bacteriology, Proteomics

## Abstract

To thrive in nature, bacteria have to reproduce efficiently under favorable conditions and persist during stress. The global strategy that integrates the growth control and stress response remains to be explored. Here, we find that a moderate induction of (p)ppGpp reduces growth rate but significantly enhances the stress tolerance of *E. coli*, resulting from a global resource re-allocation from ribosome synthesis to the synthesis of stress-responsive proteins. Strikingly, the activation of stress response by (p)ppGpp is still largely retained in the absence of RpoS. In addition, (p)ppGpp induction could activate the catabolism of alanine and arginine, facilitating the adaption of bacteria to nutrient downshift. Our work demonstrates that the activation of stress response by (p)ppGpp could occur in an RpoS-independent manner and (p)ppGpp enables bacteria to integrate the control of growth and stress response in a seesaw fashion, thus acting as an important global regulator of the bacterial fitness landscape.

## Highlights

• (p)ppGpp induction reduces growth rate but enhances the stress tolerance of *E. coli*
• (p)ppGpp induction triggers a resource re-allocation from biosynthesis to stress response
• The activation of stress response of (p)ppGpp is largely independent of RpoS
• (p)ppGpp also promotes the catabolism of alanine and arginine of *E. coli*

## Graphical abstract

**Figure:**
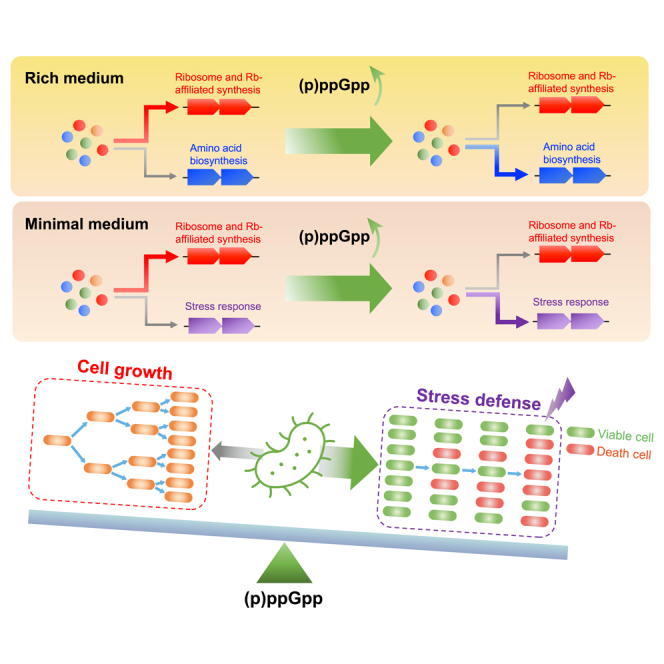

## Introduction

To thrive in nature, bacterial cells must be able to reproduce rapidly under favorable conditions and persist during adverse conditions. Unlike nutrient broth where abundant nutrient sources are provided, the nutrients are limited and highly fluctuating in the natural living environments of bacteria (e.g., intestinal tract, the natural nich of *E. coli*).^1^^,^^2^^,^^3^^,^^4^^,^^5^^,^^6^^,^^7^ Even worse, bacterial cells are frequently exposed to various types of abiotic stresses such as hyperosmotic stress, low pH, oxidative stress, cold/heat shock, and antibiotic treatment.^8^^,^^9^^,^^10^^,^^11^^,^^12^^,^^13^ Since growth control and stress response are the two most fundamental processes of bacteria, it is conceivable that bacteria have evolved sophisticated molecular strategies to balance population growth and stress response in order to maximize fitness in highly fluctuating environments. However, growth control^14^^,^^15^^,^^16^ and stress response of bacteria^9^^,^^13^ have largely been studied separately in historical views; it remains poorly understood regarding the molecular strategy of bacteria to integrate the control of these two global processes.

Two global signaling pathways lie at the core of bacterial response to adverse conditions: the stringent response mediated by guanosine tetraphosphate and pentaphosphate, [(p)ppGpp]^17^^,^^18^^,^^19^^,^^20^ and the general stress response mediated by the $\sigma^{s}$ (RpoS) subunit of RNA polymerase.^9^^,^^13^^,^^21^^,^^22^^,^^23^ Both stringent response and general stress response can be trigged by nutrient starvation (stationary phase) and a variety of abiotic stresses (e.g., osmotic stress, oxidative stress, and low pH), reshaping the global gene expression pattern of bacterial cells by affecting the expressions of hundreds of genes,^9^^,^^17^^,^^18^^,^^20^^,^^21^ further enhancing the bacterial tolerance to various environmental stressors and antibiotic treatment.^24^^,^^25^^,^^26^^,^^27^ The drastic accumulation of (p)ppGpp during stringent response causes complete shutdown of various central dogma processes (replication, rRNA transcription, ribosome maturation, and translation).^17^^,^^28^ (p)ppGpp is also involved in the metabolic control of bacterial cells via e.g., activating amino acid biosynthesis,^18^^,^^29^ allowing bacterial cells to more quickly adapt to sudden nutrient downshift.^6^^,^^30^ In comparison, the effect of RpoS is more specific to stress response and management; its target gene products are directly involved in protecting bacterial cells against various types of abiotic and biotic stresses.^9^^,^^21^^,^^31^ Interestingly, there exists a tight link between stringent response and general stress response since (p)ppGpp could positively regulate the expression of RpoS via direct transcription activation and inhibition of its proteolysis through increasing the level of anti-adaptor protein IraP.^9^^,^^17^^,^^21^^,^^32^ Therefore, an intriguing scenario is that during stringent response, (p)ppGpp stimulates the expression of RpoS, further activating the general stress response to facilitate the survival of bacteria. In support of this, it has been found that the expression of RpoS is severely impaired and delayed in the ppGpp^0^ (Δ *relA* Δ *spoT*) background after entry into stationary phase.^21^^,^^33^

It has been proposed that the regulation of stress response and amino acid biosynthesis might require different threshold levels of (p)ppGpp.^34^^,^^35^ The effect of (p)ppGpp on stress response (via RpoS) might require a very high threshold level of (p)ppGpp, occurring during stringent response when (p)ppGpp could be dramatically stimulated by dozens of folds.^6^^,^^36^ Instead, basal levels of (p)ppGpp during exponential growth are enough to control amino acid biosynthesis (e.g., via Lrp protein).^34^^,^^35^ It has been proposed that the physiological function of basal levels of (p)ppGpp during exponential growth could be different from high levels of (p)ppGpp during stringent response.^17^^,^^37^ For example, recent studies have demonstrated that basal levels of (p)ppGpp are involved in regulating bacterial exponential growth via modulating proteome allocation under different nutrient conditions.^38^^,^^39^^,^^40^^,^^41^^,^^42^^,^^43^ From rich conditions to poor conditions, the cellular pools of (p)ppGpp increase only moderately by several folds, inhibiting the ribosomes synthesis but increasing the expression of metabolic proteins (e.g., amino acid biosynthesis) to help bacteria adapt to poor nutrient conditions.^6^^,^^7^^,^^38^^,^^39^ Nevertheless, the aforementioned notion remains to be validated due to a lack of genome-wide picture of the regulation of stress response by (p)ppGpp. Specially, it remains unclear whether the activation of bacterial stress response by (p)ppGpp is just via RpoS or could also occur in an RpoS-independent manner. Insight into these questions is crucial in understanding the architecture of stress response system and principles of resource allocation inside bacterial cells.

Using high-coverage quantitative proteomics, here we present a genome-wide study of the regulation of cellular resource allocation by (p)ppGpp in both wild-type and *rpoS*-null *E. coli* growing in minimal medium. We show that a moderate increase in the basal level of (p)ppGpp is enough to cause slowdown of growth but significantly activate the stress response of *E. coli*, further strongly enhancing the bacterial tolerance against a variety of abiotic stresses. Moreover, the activation of stress response by (p)ppGpp is still largely retained in the absence of RpoS. Our work thus demonstrates that (p)ppGpp signaling enables bacteria to integrate the control of growth and stress response in a seesaw fashion.

## Results

### Proteome allocations of *E. coli* growing in rich medium and minimal medium

**Figure 1:**
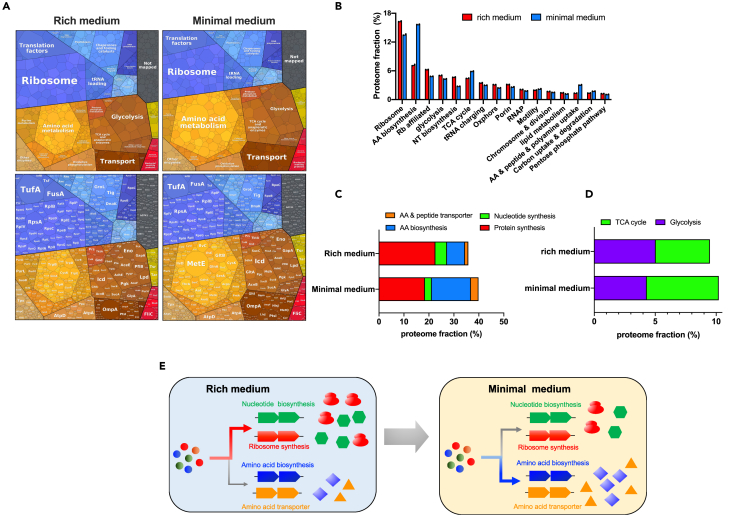
The proteome allocations of wild-type *E. coli* strain in rich medium and minimal medium Data of rich medium are from AE155 project in Zhu & Dai^6^ while data of glucose minimal medium are from RE198 project in this work. (A) The proteome allocation of *E. coli* analyzed by proteomaps website. (B) The mass fractions of various proteome sectors. (C) The mass fractions of four proteome sectors including amino acid (AA) biosynthesis, AA and peptide transporters, nucleotide biosynthesis, and protein synthesis. The sector of protein synthesis includes both ribosomal proteins and ribosome-affiliated proteins such as EF-Tu, EF-G. (D) The mass fractions of TCA cycle versus glycolysis. (E) The resource allocation strategy of *E. coli* during growth in rich medium and minimal medium. The rich medium refers to glucose medium supplemented with casamino acids (cAA) while the minimal medium refers to glucose minimal medium.

In the natural niches of bacteria, the nutrient conditions are often fluctuating and limited. Therefore, we first explored the differences in the proteome allocation modes of *E. coli* cells during growth in amino acid-supplemented rich medium and glucose minimal medium. The high-coverage 4D label-free mass spectrometry approach captured over 2,500 proteins of *E. coli* proteome (Table S1) with high reproducibility (Figures S1 and S2). Analysis with proteomap website (https://proteomaps.net)^44^ provided a direct visualization of the proteome allocation of *E. coli* (Figure 1A). From rich medium (glucose plus casamino acids medium) to glucose minimal medium, we found a strong decrease in the levels of ribosomal proteins and translation-affiliated proteins but a strong increase in the levels of amino acid biosynthetic proteins and transporters (Figure 1A). Going beyond visualization, we next quantified the proteome fractions of various functional sectors (Figure 1B). Being consistent with the proteomap analysis, the major changes of proteome allocation of *E. coli* from rich medium to minimal medium include a strong downregulation of protein synthesis machinery and nucleotide biosynthesis (coordinates with the lower demand of rRNA synthesis)^45^ but a strong upregulation of amino acid biosynthesis and transporter systems (Figure 1C), being consistent with previous reports.^7^^,^^46^ Such a proteome allocation strategy allows *E. coli* to meet the high demand of *de novo* amino acid biosynthesis in minimal medium at the expense of ribosome synthesis (Figure 1E). In addition, both proteomap analysis (Figure 1A) and absolute quantification (Figure 1D) show a higher TCA cycle over glycolysis ratio from rich medium to minimal medium, suggesting the requirement for a higher efficiency of energy generation and carbon utilization.^4^^,^^47^

### Proteome allocation of *E. coli* during (p)ppGpp overproduction in minimal medium

**Figure 2:**
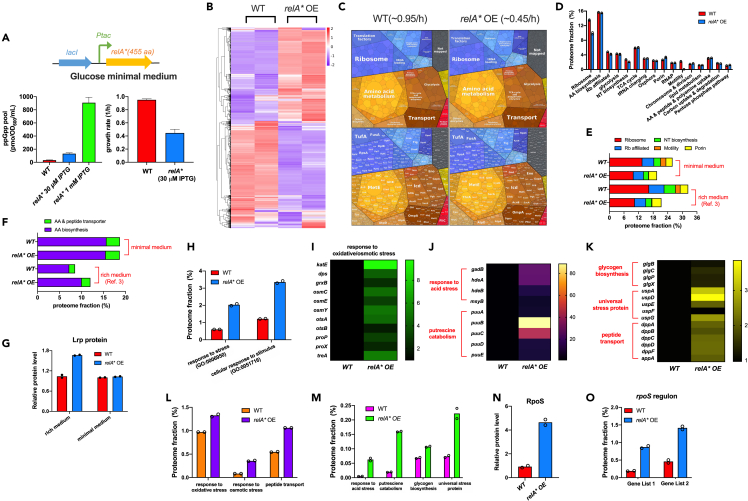
Global effect of (p)ppGpp overproduction on proteome resource allocation of *E. coli* (A) The effect of RelA∗ overexpression (OE) on the growth rates and cellular ppGpp pools of *E. coli* in glucose minimal medium. Data are represented as mean ± SD. RelA∗ protein consists of the N-termini 455 amino acid residues of native RelA protein with constitutive (p)ppGpp synthetase activity. (B) Heatmap analysis of the proteome abundances of wild type strain and RelA∗ OE strain (IPTG: 30 μM). (C) The proteome resource allocation of *E. coli* wild type strain and RelA∗ OE strain analyzed by proteomaps website. (D) The mass fractions of various proteome sectors. (E) The mass fractions of five proteome sectors including ribosome synthesis, ribosome-affiliated proteins, nucleotide (NT) biosynthesis, motility, and porin. (F) The mass fractions of amino acid (AA) biosynthesis sector and AA & peptide transporter sector. (G) The relative abundance of leucine-responsive protein, Lrp, a key regulator of AA biosynthesis. (H) The mass fractions of two GO-ontology enriched groups: “response to stress” (GO term: 0006950) and “cellular response to stimulus” (GO term: 0051716), see the gene list in Table S4. (I–K) The relative abundances of individual stress-responsive proteins. (L–M) The proteome fractions of various stress-responsive proteins. (N) The relative abundance of RpoS protein. (O) The proteome fractions of *rpoS*-regulon genes. Gene list 1 and Gene list 2 of *rpoS*-regulon were based on Patten et al. and Weber et al., respectively.^48^^,^^49^ See Table S4. The rich medium refers to glucose medium supplemented with casamino acids (cAA). The minimal medium refers to glucose minimal medium. Data of rich medium shown in 2E, 2F, and 2G are taken from the AE155 project data provided in Zhu & Dai^6^ while data of glucose minimal medium are from RE198 project in this work.

We next explored the effect of (p)ppGpp overproduction on the growth physiology of *E. coli* in glucose minimal medium. (p)ppGpp overproduction was achieved by overexpressing the constitutively active (p)ppGpp synthetase, RelA∗ protein (Figure 2A).^6^^,^^29^^,^^40^ At a low concentration of IPTG inducer (30 μM), (p)ppGpp level exhibited a moderate (4- to 5-fold) increase (Figure 2A, left); meanwhile, the exponential growth was still sustained but decreased by over 50% compared with wild-type strain (Figure 2A, right). Addition of 1 mM IPTG led to an extremely high level of (p)ppGpp (Figure 2A, left), which was comparable to the level during stringent response.^6^^,^^36^

We then compared the proteome allocation of *E. coli* wild-type strain (λ:0.95/h) and RelA∗ overexpression (OE) strain (with 30 μM IPTG, λ:0.45/h) during growth in glucose minimal medium (Table S1). Heatmap analysis shows that the (p)ppGpp overproduction substantially re-shapes the global gene expression pattern of *E. coli* (Figure 2B). Proteomap analysis shows that (p)ppGpp overproduction lowers the level of ribosome (Figure 2C), which could naturally explain the slowdown of exponential growth and was similar to the effect of (p)ppGpp overproduction on cells growing in rich medium as shown recently.^6^ In addition, we also noted a slight upregulation of porin proteins but a strong downregulation of ribosome and ribosome (Rb)-affiliated proteins, nucleotide biosynthetic proteins, and motility proteins during increased (p)ppGpp level (Figures 2D and 2E; Table S3). However, in contrast to the case of (p)ppGpp induction in rich medium, there is no marked increase in amino acid (AA) biosynthesis sector during (p)ppGpp induction in minimal medium (Figures 2F; Table S3). Moreover, the level of Lrp protein (leucine-responsive protein), a global regulator of amino acid biosynthesis in *E. coli*,^50^^,^^51^ exhibits a similar trend to the level of AA biosynthesis and transporter sectors (Figure 2G).

We next wondered what kinds of proteins were upregulated by (p)ppGpp induction in minimal medium and thus did a Gene Ontology (GO) analysis of those individual upregulated proteins. We found that individual proteins belonging to “response to stress” group (GO:000695) and “cellular response to stimulus” group (GO:0051716) were significantly enriched (Figure 2H), demonstrating that (p)ppGpp overproduction substantially stimulates the stress response of *E. coli* (Table S4). The expressions of many proteins involved in response to oxidative/acid/osmotic stress, the universal stress proteins,^52^ as well as glycogen biosynthesis (related to carbon and energy storage during nutrient limitation)^53^ were significantly upregulated (Figures 2I–2M). In addition, proteins related to putrescine catabolism^54^^,^^55^ and small peptide transport^56^ are also upregulated (Figures 2J and 2K). Importantly, we noted that the level of RpoS increased nearly 5-fold and the RpoS regulon was strongly upregulated (Figures 2N and 2O; Table S5),^48^^,^^49^ providing an intuitively reasonable explanation of the activation of stress response by (p)ppGpp. Taken together, these results show that (p)ppGpp overproduction triggers a proteome resource re-allocation from ribosome biosynthesis to stress response.

### (p)ppGpp overproduction enhances stress tolerance of *E. coli*

**Figure 3:**
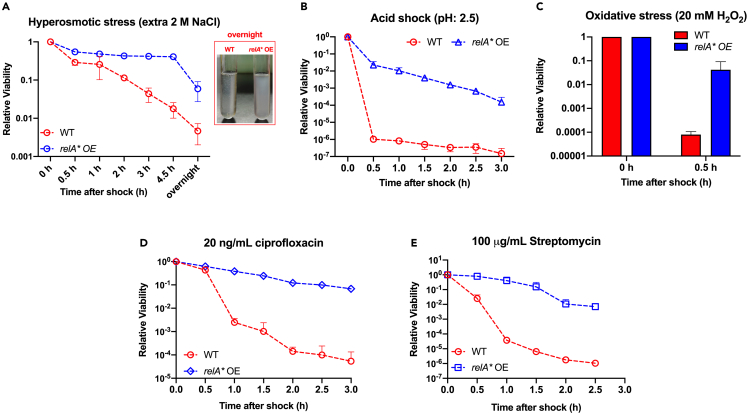
Effect of (p)ppGpp overproduction on the tolerance of *E. coli* against various abiotic stresses (A) Hyperosmotic shock triggered by extra 2 M NaCl. The small photo shows the overnight culture after hyperosmotic shock. Large amounts of agglomerated debris appeared in the overnight liquid culture of wild-type strain while the overnight liquid culture of RelA∗ OE strain was still normal. (B) Acid stress trigged by transition to EG medium (pH: 2.5). (C) Oxidative stress trigged by addition of a lethal dose (20 mM) of hydrogen peroxide. (D) Treatment by a lethal dose (20 ng/mL) of ciprofloxacin. (E) Treatment by a lethal dose (100 μg/mL) of streptomycin. For the cases above, the viability at 0 h is set as 1. Data are represented as mean ± SD.

A global upregulation of the stress response pathways should in principle protect bacteria against various abiotic stresses. To verify this notion, we next had *E. coli* been subjected to various kinds of environmental stressors including hyperosmotic stress (extra 2 M NaCl), acid stress (pH 2.5), oxidative stress (20 mM hydrogen peroxide), and antibiotic treatment (20 ng/mL ciprofloxacin or 100 μg/mL streptomycin) and then measured the time-course viability (the relative CFU/OD) of *E. coli* cells. Strikingly, a moderate induction of (p)ppGpp (the RelA∗ OE strain with 30 μM IPTG) is enough to substantially increase the survival of *E. coli* cells in all cases of stress tested here (Figure 3). Take hyperosmotic shock as the example, the wild-type strain exhibited a rapid loss of viability during the initial 4.5 h after shock (over 95% loss in 4.5 h) while the viability of RelA∗ OE strain was largely maintained during the same period (Figure 3A). Moreover, large amounts of agglomerated debris appeared in the overnight liquid culture of wild-type strain while the overnight liquid culture of RelA∗ OE strain was still normal (the small photo of Figure 3A). We also noted a substantial increase in the antibiotic tolerance of *E. coli* during (p)ppGpp overproduction, which is consistent with the notion that (p)ppGpp is a positive regulator of bacterial antibiotic tolerance (Figures 3D and 3E).^11^^,^^24^^,^^57^^,^^58^ Nevertheless, the increased antibiotic tolerance here is observed during exponential growth stage instead of nutrient starvation or stationary phase as previously studied,^11^^,^^57^ suggesting that nutrient depletion is not a prerequisite for higher drug tolerance. Collectively, these results show that although (p)ppGpp overproduction lowers the growth rate of *E. coli* on one side, it strongly promotes the survival of bacteria during stressful conditions on the other side.

### Activation of stress response by (p)ppGpp overproduction is retained in *rpoS*-null background

**Figure 4:**
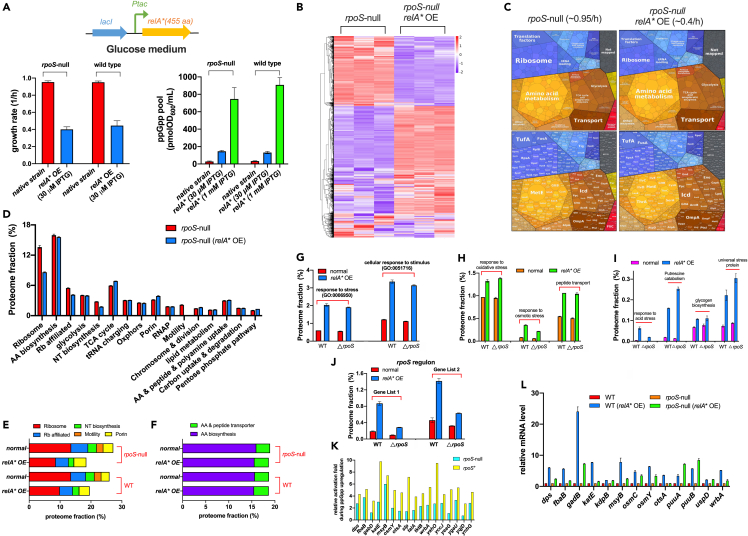
Global effect of (p)ppGpp overproduction on proteome resource allocation of *E. coli rpoS*-null strain (A) The effect of RelA∗ overexpression (OE) on the growth rates and cellular ppGpp pools of *E. coli* wild type strain and *rpoS*-null strain in glucose minimal medium. Data are represented as mean ± SD. (B) Heatmap analysis of the proteome abundances of *rpoS*-null strain and its RelA∗ OE strain (IPTG: 30 μM). (C) The proteome allocations of *rpoS*-null strain and its RelA∗ OE strain analyzed by proteomaps website. (D) The mass fractions of various proteome sectors. Data are represented as mean ± SD. (E) The mass fractions of five functional sectors including ribosome synthesis, ribosome-affiliated proteins, nucleotide (NT) biosynthesis, motility, and porin. (F) The mass fractions of amino acid (AA) biosynthesis sector and AA & peptide transporter sector. (G–I) The mass fractions of various groups of stress-responsive proteins, see the gene list in Table S9. Data are represented as mean ± SD. (J) The proteome fractions of genes belonging to *rpoS*-regulon. Gene list 1 and Gene list 2 of *rpoS*-regulon were based on Patten et al. and Weber et al*.*^48^^,^^49^ See Tables S5 and S10. Data are represented as mean ± SD. (K) The relative activation folds of expressions of typical *rpoS*-regulon genes during (p)ppGpp overproduction in both wild-type and *rpoS*-null background. (L) The relative mRNA levels of some typical stress-responsive genes during (p)ppGpp overproduction in both wild-type and *rpoS*-null backgrounds. Data are represented as mean ± SD.

**Figure 5:**
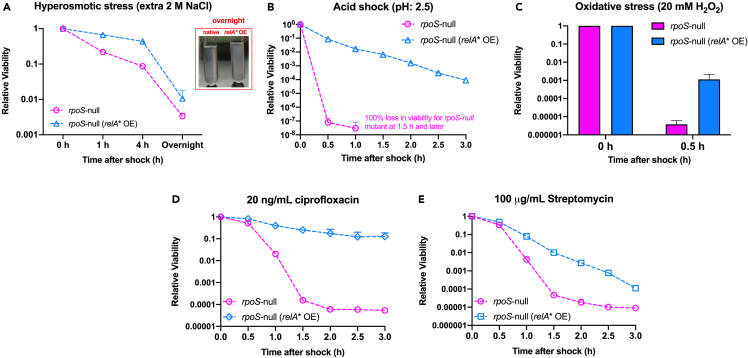
Effect of (p)ppGpp overproduction on the tolerance of *E. coli rpoS*-null strain against various abiotic stresses (A) Hyperosmotic shock triggered by extra 2 M NaCl. Same as Figure 3A, the small photo shows the overnight culture after hyperosmotic shock. Large amounts of agglomerated debris also emerged in the overnight liquid culture of native *rpoS*-null strain while the overnight liquid culture of *rpoS*-null RelA∗ OE strain was still normal. (B) Acid stress trigged by transition to EG medium (pH: 2.5). (C) Oxidative stress trigged by addition of a lethal dose (20 mM) of hydrogen peroxide. (D) Treatment by a lethal dose (20 ng/mL) of ciprofloxacin. (E) Treatment by a lethal dose (100 μg/mL) of streptomycin. For all the cases above, the viability at 0 h is set as 1. Data are represented as mean ± SD.

Mechanistically, the activation of stress response by (p)ppGpp overproduction could be simply attributed to its activation of RpoS (Figure 2N). If so, the activation of stress response should largely disappear in the absence of RpoS. To investigate this issue, we again investigated the proteomes of *E. coli rpoS*-null strain during (p)ppGpp overproduction in minimal medium (Table S6). The *rpoS*-null strain and wild-type strain had similar growth rates in glucose minimal medium (red bar, left panel of Figure 4A). Moreover, RelA∗ OE at IPTG 30 μM also moderately increased the cellular (p)ppGpp level but reduced the growth rate of *rpoS-*null strain, being comparable to its effect on wild-type strain (Figure 4A). Proteomic studies demonstrated that (p)ppGpp overproduction also re-shaped the global gene expression pattern of *rpoS-*null strain (Figure 4B) as observed for wild-type strain (Figure 2B). Proteomap visualization (Figure 4C) and absolute quantification (Figures 4D and 4E; Tables S7 and S8) with iBAQ intensity again show that the levels of ribosome, ribosome (Rb)-affiliated proteins, nucleotide biosynthesis, and motility proteins strongly drop with increased (p)ppGpp level (Table S8). In contrast, there were no marked differences between wild-type strain and *rpoS*-null strain in the abundances of various metabolic sectors (Figure S3), being consistent with their similar exponential growth rates in glucose minimal medium (Figure 4A). Moreover, being similar to the case of wild-type strain, (p)ppGpp overproduction does not upregulate the levels of amino acid biosynthetic proteins in *rpoS*-null background as well (Figure 4F). We then turned to those stress-responsive proteins which are upregulated by (p)ppGpp overproduction in wild-type background (Figures 2H–2M). Strikingly, the levels of “response to stress” sector (GO:000695) and “cellular response to stimulus” sector (GO:0051716) (the same gene list as Figure 2H) are still strongly upregulated by (p)ppGpp overproduction for *rpoS*-null strain, with the absolute abundance being comparable to that of wild-type background (Figures 4G; Table S9). Similar results were found for various specific stress-responsive sectors including oxidative, acid, osmotic shock response, the universal stress proteins, glycogen biosynthesis, putrescine catabolism, and peptide transport (Figures 4H and 4I; Table S9). For those genes belonging to *rpoS*-regulon,^48^^,^^49^, (p)ppGpp overproduction could still activate the expressions of them in *rpoS*-null strain, albeit with a lower effect than that in wild-type strain (Figures 4J and 4K; Table S10). We further measured the relative mRNA levels of a dozen of typical stress-responsive genes belonging to osmotic stress response (*kdpB*, *otsA*, *osmC*, *osmY*), oxidative stress response (*dps, katE*), acid stress response (*gadB*, *msyB*), putrescine catabolic process (*puuA*, *puuB*), and universal stress protein (*uspD*). Being consistent with the proteomic results, the mRNA levels of all these genes were strongly upregulated in wild-type background during (p)ppGpp induction (blue bar versus red bar in Figure 4L). Meanwhile, for some typical genes of *rpoS*-regulon (*dps*, *fbaB*, *katE*, *msyB*, *osmY*, *otsA*, *wrbA*, *osmC*, *gadB*; see Ecocyc database^59^), the stimulations of their expressions by (p)ppGpp induction were more or less compromised in *rpoS*-null strain (green bar versus orange bar, Figure 4L), but were still retained. For other genes such as *puuA*, *puuB*, *uspD*, and *kdpB*, the stimulations of their expressions by (p)ppGpp induction were not negatively affected in the absence of RpoS. Therefore, it is likely that (p)ppGpp mainly activates stress response at the transcription level, depending only partially on RpoS. We further found that the increased (p)ppGpp levels also strongly enhanced the tolerance of *rpoS*-null strain to a variety of abiotic stresses including hyperosmotic stress, acid stress, oxidative stress, and drug treatment (Figure 5). Collectively, these results strongly support that the (p)ppGpp-mediated activation of stress response for *E. coli* could occur in an RpoS-independent manner.

**Figure 6:**
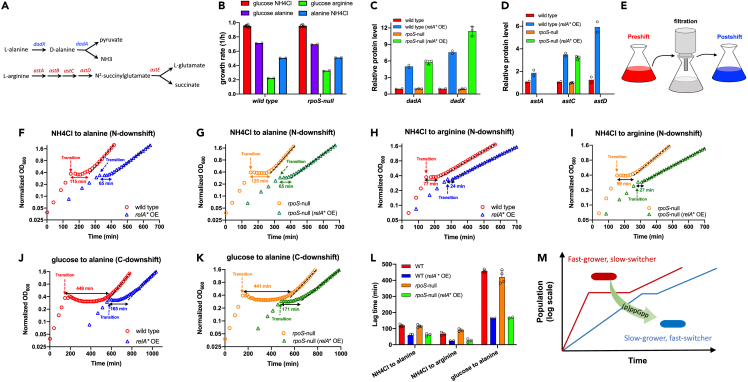
(p)ppGpp induction promotes the adaption of *E. coli* to nutrient downshift via activating alanine and arginine catabolism The *relA*∗ OE condition denotes wild type RelA∗ OE and *rpoS*-null RelA∗ OE strains growing in glucose+NH_4_Cl medium supplemented with 30 μM IPTG as detailed in Figures 2 and 4, respectively. (A) Alanine and arginine catabolism in *E. coli*. (B) The growth rates of wild-type strain and *rpoS*-null strain in various minimal media supplemented with alanine or arginine as the nitrogen or carbon source. Data are represented as mean ± SD. (C) The relative abundances of the key proteins involved in alanine catabolism. Data are represented as mean ± SD. (D) The relative abundances of the key proteins involved in arginine catabolism. Data are represented as mean ± SD. (E) Schematics of the nutrient downshift procedure. (F) Growth and lag of wild-type strain and its *relA*∗ OE strain during nitrogen (N) downshift from NH_4_Cl to alanine. (G) Growth and lag of *rpoS*-null strain and its *relA*∗ OE strain during N-downshift from NH_4_Cl to alanine. (H) Growth and lag of wild-type strain and its *relA*∗ OE strain during N-downshift from NH_4_Cl to arginine. (I) Growth and lag of *rpoS*-null strain and its *relA*∗ OE strain during N-downshift from NH_4_Cl to arginine. (J) Growth and lag of wild-type strain and its *relA*∗ OE strain during carbon (C) downshift from glucose to alanine. (K) Growth and lag of *rpoS*-null strain and its *relA*∗ OE strain during C-downshift from glucose to alanine. (L) The lag times of wild-type strain, *rpoS*-null strain and their *relA*∗ OE strains in all the three nutrient downshift experiments. Data are represented as mean ± SD. (M) Schematics: (p)ppGpp induction reduces the steady-state growth rate of bacteria but promotes the bacterial adaption to nutrient downshift, thus transforming the bacteria from fast-grower & slow-switcher to slow-grower & fast switcher.

Besides all the stresses mentioned previously, a very common challenge that bacteria undergo in nature is adapting to changing nutrient environments (e.g., famine-feast cycle in the mammalian intestine).^5^ In addition to the commonly used glucose (the preferred carbon source) and NH_4_Cl (the preferred nitrogen source), some amino acids could serve as alternative carbon or nitrogen sources for gut bacteria like *E. coli*.^60^ For example, *E. coli* has alanine and arginine catabolism pathways (Figure 6A), being able to utilize alanine as the sole carbon or nitrogen source and utilize arginine as the sole nitrogen source.^60^ As nitrogen sources, alanine and arginine supported lower growth rates of *E. coli* than NH_4_Cl (glucose as the carbon source, Figure 6B). Meanwhile, as a carbon source, alanine supported a lower growth rate than glucose (NH_4_Cl as the nitrogen source, Figure 6B). Strikingly, proteomic studies showed that (p)ppGpp induction strongly upregulated the alanine and arginine catabolism pathways of *E. coli* when growing in glucose+NH_4_Cl medium (Figures 6C and 6D), raising the possibility that (p)ppGpp can accelerate the bacterial adaption to amino acid-contained environments. To verify this scenario, we performed three types of nutrient downshift experiments (Figure 6E and STAR Methods): two nitrogen (N) downshift experiments (NH_4_Cl shifted to alanine; NH_4_Cl shifted to arginine) with glucose as carbon source and one carbon (C) downshift experiment (glucose shifted to alanine) with NH_4_Cl as the nitrogen source. Indeed, in all the three types of nutrient downshift, (p)ppGpp induction substantially reduced the growth lag by a factor of 2–3 in both wild-type background (blue versus red symbols in Figures 6F, 6H, and 6J) and *rpoS*-null background (green versus orange symbols in Figures 6G, 6I, and 6K), as summarized in Figure 6L. Taken together, these results show that (p)ppGpp induction reduces the growth rate of bacteria while promotes the bacterial adaption to nutrient downshift, further transforming the bacteria from a faster-grower & slow-switcher to a slow-grower & faster switcher and thus leading to a trade-off between growth rate and adaption to nutrient downshift (Figure 6M).

## Discussion

**Figure 7:**
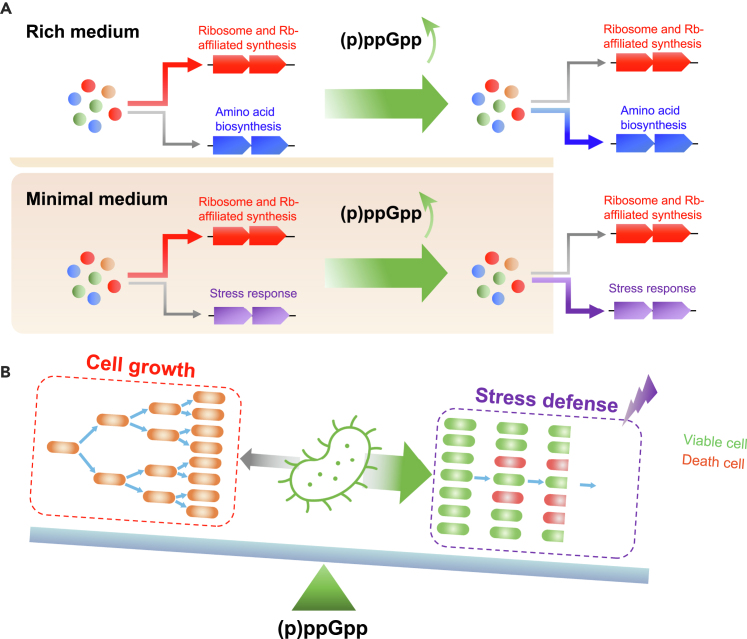
(p)ppGpp manages proteome resource allocation in a seesaw fashion (A) In rich medium, (p)ppGpp overproduction triggers a proteome resource re-allocation from ribosome synthesis to amino acid biosynthesis. In minimal medium, (p)ppGpp overproduction triggers a proteome resource re-allocation from ribosome synthesis to stress response. Such proteome resource re-allocation could occur in an RpoS-independent manner. (B) (p)ppGpp simultaneously controls cell growth and stress defense in a seesaw fashion. A moderate induction in the cellular (p)ppGpp level is enough to significantly enhance bacterial stress defense at the expense of sacrificing exponential growth.

Rapid growth under favorable conditions and defense against stress under harsh environments are two most fundamental properties of bacteria to thrive in nature. However, the molecular strategy that balances cell growth and stress response remains less well defined for bacteria. In rich medium, it has been found that (p)ppGpp overproduction inhibits cell growth but accelerates the growth adaption of *E. coli* to amino acid downshift via triggering resource re-allocation from ribosome synthesis to amino acid biosynthesis (Figure 7A).^6^^,^^29^ Here we find that, in minimal medium, (p)ppGpp overproduction causes slowdown of cell growth but enhances the tolerance of bacteria to multiple abiotic stresses via triggering resource re-allocation from ribosome synthesis to stress response (Figure 7A). Therefore, (p)ppGpp signaling enables bacteria integrate the control of cell growth and stress response in a seesaw fashion, namely, enhancing one trait at the expense of sacrificing another trait (Figure 7B). Perturbing the cellular (p)ppGpp pools could reset the balance between stress resistance and growth of bacterial cells.

Our study has also demonstrated that (p)ppGpp is involved in the regulation of not only amino acid biosynthesis but also amino acid catabolism (degradation). The activation of amino acid biosynthesis by (p)ppGpp induction is observed in rich medium but not in minimal medium here where the expression of amino acid biosynthesis pathway has already been fully activated (Figure 2F). In contrast, (p)ppGpp induction could stimulate the expressions of alanine and arginine catabolism pathways in minimal media, thus facilitating the utilization of amino acids as alternative nitrogen or carbon sources. Therefore, our result further broadens the physiological functions of (p)ppGpp in regulating bacterial metabolism. Such a type of regulatory strategy could be of important ecological meaning as it could enable *E. coli* to achieve a higher efficiency of assimilating alternative carbon/nitrogen sources existing in their natural niches. In addition, recent studies have shown that growth rate and adaption to nutrient transitions are both important traits that could affect the fitness of bacteria in their ecological niches.^4^^,^^61^^,^^62^ These two traits often have a trade-off relation in bacterial cells. Faster-grower & slow-switcher and slow-grower & faster switcher could often stably co-exist in ecological environments.^61^^,^^62^ Our study shows that (p)ppGpp induction reduces the growth rate of bacteria but promotes the bacterial adaption to nutrient downshift, further transforming the bacteria from fast-grower & slow-switcher to slow-grower & fast switcher. Therefore, perturbing (p)ppGpp signaling could be a plausible molecular strategy adopted by bacteria to achieve different traits in order to adapt to different types of natural environments.

It has been found that during nutrient starvation, Lrp-regulated amino acid biosynthetic enzymes could be induced earlier by (p)ppGpp than RpoS-dependent stress-responsive genes, which could be attributed to that stress-responsive genes require a much higher threshold level of (p)ppGpp (>400 pmol/mL/OD in stringent response which leads to complete growth arrest) than metabolic proteins.^34^^,^^35^ Here, we find that actually a moderate increase in the basal level of (p)ppGpp (no higher than 150 pmol/mL/OD), which still permits a moderate exponential growth rate, is enough to strongly stimulate the stress response of *E. coli* and further enhance its stress tolerance. Counterintuitively, the activation of stress response by (p)ppGpp could largely occur in an RpoS-independent manner, suggesting the existence of a more general regulatory mechanism by (p)ppGpp via modulating cellular resource allocation. A possible underlying picture could be that: in rich medium, the activation of amino acid promoter by (p)ppGpp could be via either direct effect on RNA polymerase (RNAP) in combination with DksA or indirect effect resulting from releasing RNAP from rRNA promoters.^18^ In the case of minimal medium, the amino acid promoters have already been saturated by RNA polymerase due to the release of local transcription repression so that (p)ppGpp induction could not further activate them, and instead, a relatively larger fraction of free RNA polymerases (from the release of rRNA promoters as well) could be available for transcribing those basal-level stress-responsive genes. In this picture, (p)ppGpp could activate the bacterial stress response in two ways: direct activation via RpoS and indirect activation via modulating resource re-allocation from inhibiting ribosome synthesis (Figure 7A). It is also possible that there exist various other indirect or direct mechanisms considering the various *in vivo* targets of (p)ppGpp.^18^^,^^27^^,^^63^

The integrated control of cell growth and stress response by (p)ppGpp could be of both theoretical and practical values. Perturbing cellular (p)ppGpp levels allows artificial manipulation of the fitness landscape of bacteria between rapid growth and stress defense. In the highly fluctuating living habitat of bacterial cells, combating stress instead of rapid growth could more likely be the primary “concern” for those natural bacterial isolates.^64^ For example, pathogens need to defend themselves against various biotic (attack by immune systems) and abiotic stresses (low pH, oxidative stress, and drug treatment) during infecting their hosts.^10^ In such case, (p)ppGpp signaling pathway could be employed by pathogens to become more stress-resistant in order to survive better inside the host. From this view, targeting the (p)ppGpp pathway may act as an effective auxiliary approach to enhance the efficacy of antimicrobial therapy. In addition, the governing principles of growth control and stress response could guide the synthetic-biology design of intelligent engineered cells for special applications.

### Limitation of the study

We point out here that the detailed RpoS-independent mechanism of (p)ppGpp in activating various stress-responsive genes remains to be explored in future studies.

## STAR★Methods

### Key resources table

**Table:** 

REAGENT or RESOURCE	SOURCE	IDENTIFIER
**Bacterial and virus strains**		

*E. coli NCM3722 K-12* strain	Lab stock	N/A
*E. coli NCM3722 rpoS-null* strain	Terry Hwa lab (UCSD)	N/A
*E. coli NCM3722* RelA∗ OE strain	This study	N/A
*E. coli rpoS-null* RelA∗ OE strain	This study	N/A

**Chemicals, peptides, and recombinant proteins**		

NH_4_Cl	Sigma	G5767
glucose	Sigma	213330
alanine	Aladdin (Shanghai)	CAS: 56-41-7
arginine	Aladdin (Shanghai)	CAS: 74-79-3
LB broth	Coolaber (Beijing)	PM0010
ampicillin	Coolaber (Beijing)	CA2031
IPTG (Isopropyl β-D-thiogalactoside)	GLPBIO	GC30002
6-azauracil	Aladdin (Shanghai)	A124269
ppGpp (guanosine-3′,5′-bisdiphosphate)	TriLink	N-6001
ciprofloxacin	Sigma	CAS: 85721-33-1
streptomycin	Coolaber (Beijing)	CS10481
MgSO_4_·7H_2_O	Aladdin (Shanghai)	CAS: 10034-99-8
K_2_HPO_4_	Aladdin (Shanghai)	CAS: 7758-11-4
NaNH_4_HPO_4_	Aladdin (Shanghai)	CAS: 7783-13-3
citrate	Aladdin (Shanghai)	CAS: 5959-29-1
KH_2_PO_4_	Aladdin (Shanghai)	CAS: 7778-77-0
phosphoric acid	Merck	49685
acetonitrile	Aladdin (Shanghai)	A104440
tetrabutylammonium phosphate	TCI	CAS:5574-97-0

**Critical commercial assays**		

Total RNA extraction kit	TianGen	DP430
First-strand cDNA synthesis reverse transcriptase kit	TianGen	KR118
Plasmid extraction kit	TianGen	DP103
Bacterial genome extraction kit	TianGen	DP302
Hieff® qPCR SYBR Green Master Mix (Low Rox Plus)	Yeasen Biotech	11202ES03

**Recombinant DNA**		

pLAS13	Richard Gourse lab	N/A

**Deposited data**		

The proteomic datasets generated in this study have been deposited to the ProteomeXchange Consortium (http://www.proteomexchange.org/) via the PRIDE repository.	This paper	PXD042250

### Resource availability

#### Lead contact

Further information and requests for materials should be directed to and will be fulfilled by the lead contact, Xiongfeng Dai (daixiongfeng@ccnu.edu.cn).

#### Materials availability

This study did not generate unique reagents.

#### Data and code availability

All data reported in this paper will be available from the lead contact upon reasonable request. The raw data of proteomics are publicly available as of the data of the publication in the PRIDE repository. The identifier is listed in the key resources table.
This paper does not report original code.
Any additional information required to reanalyze the data reported in this paper is available from the lead contact upon reasonable request.

### Experimental model and subject participant details

Wild type *E. coli NCM3722 K-12* strain and its derivatives were used in this study. For all related experiments, we always prepared fresh LB plates of *E. coli* cells (from the glycerol stock of −80°C freezer) as the starting material.

### Method details

#### Strains

Strains used in this study were derivatives of *E. coli* K-12 NCM3722 strain including wild type strain, its *rpoS*-null strain (NQ1191, kindly provided by Terry Hwa lab, UCSD)^22^ and the RelA∗ overexpression strain of both wild type background and *rpoS-*null background. The RelA∗ overexpression system is based on the pLAS13 plasmid (a gift from Richard Gourse),^6^^,^^29^ in which the *relA∗*, encoding the constitutively active RelA (p)ppGpp synthetase gene of *E. coli*, is driven by the IPTG-inducible *Ptac* promoter.

#### Medium

The growth media used in this study were either LB broth (Coolaber, Beijing) and M9 glucose minimal medium. The LB broth contains 10 g/L tryptone, 5 g/L yeast extract and 10 g/L NaCl. Recipe of M9 glucose minimal medium (cold spring harbor protocol) contained 0.2% glucose as the carbon source and 1 g/L NH_4_Cl as the nitrogen source. In the nutrient downshift experiments, 0.2% alanine was either used as the carbon source (replace glucose) or the nitrogen source (replace NH_4_Cl); in addition, 10 mM arginine was used as the nitrogen source to replace NH_4_Cl. The rich medium referred in this work denotes glucose medium plus 0.2% casamino acids.

#### Cell growth

Cell growth was performed in an air bath shaker (200 rpm) under 37°C. A three-step culturing procedure was followed: seed culture, pre-culture and final experimental culture. Seed culture step: *E. coli* cells from a fresh colony in LB agar plate (Coolaber, Beijing) were inoculated into LB broth (Coolaber, Beijing) and further cultured for several hours. Pre-culture: the seed culture was washed with M9 minimal medium and transferred into M9 minimal medium for growing overnight. Final experimental culture: overnight pre-culture was inoculated into the same minimal medium at an initial OD_600_ ≈ 0.01 to 0.02 as the final experimental culture. 5–10 OD_600_ data points during exponential phase (generally within the OD_600_ range of 0.05–0.5) were measured by a Thermo Sci Genesys 50 spectrophotometer to calculate the exponential growth rate. For *E. coli relA*∗ OE strain (pLAS13-P*tac*-*relA*∗), the IPTG (purchased from GLPBIO) inducer was supplemented only at the final culture stage (at OD_60__0_ ≈ 0.04) to induce the (p)ppGpp overproduction process. In addition, 80 μg/mL ampicillin (Coolaber, Beijing) was added to the medium of *relA*∗ OE strain throughout the whole process.

#### Bacterial viability under abiotic stress

*E. coli* cells were subject to the shock of various abiotic stresses including hyperosmotic shock, acid shock, oxidative shock and treatment by ciprofloxacin or streptomycin. The exponential cultures (wild type strain, *rpoS*-null strain or their RelA∗ OE strains with 30 μM IPTG) were first growing to OD_600_ ≈ 0.3 before subjecting to various abiotic stresses. For the hyperosmotic shock experiment, the shock medium was M9 glucose minimal medium contained an additional 2 M sodium chloride (NaCl). The exponential culture was quickly collected by a 0.22 μm filter membrane using a vacuum filtration system. The cells in the membrane were then transferred into the shock medium to initiate a time-course hyperosmotic shock experiment. At different time points after shock, serially diluted cultures were plated on solid LB agar for cell counting. For the acid shock experiment, the shock medium was EG medium (0.2% glucose, 57 mM K_2_HPO_4_, 17 mM NaNH_4_HPO_4_, 10 mM citrate, 0.8 mM MgSO_4_, adjusted to pH 2.5 by HCl).^65^ The procedure was similar as described for hyperosmotic shock. For oxidative shock, 20 mM hydrogen peroxide (H_2_O_2_) was directly supplied into the medium. Similarly, 20 ng/mL ciprofloxacin (Sigma) or 100 μg/mL streptomycin (Coolaber, Beijing) was directly supplemented into the medium for antibiotic treatment.

#### mRNA level determination by qRT-PCR

Measurement of mRNA levels was based on qRT-PCR method as described in Zhu & Dai.^6^ 0.9 mL cell culture was transferred to a 1 mL pre-chilled fixed solution (60% ethanol, 2% phenol and 10 mM EDTA) and then subject to RNA extraction. Total cellular RNA of *E. coli* was then extracted using a bacterial RNA extraction kit (TianGen Biotech, Beijing). The cDNA synthesis was further performed with the first-strand cDNA synthesis reverse transcriptase kit (TianGen). The qRT-PCR reaction was performed by an ABI QuantStudio 3 real-time Thermocycler using the Hieff qPCR SYBR Green Master Mix (Yeasen Biotech) according to the manual.

#### Nutrient downshift experiments

The procedures of nutrient downshift experiments were similar as described in Zhu & Dai.^6^ 20 mL *E. coli* culture was first grown exponentially in glucose+NH_4_Cl medium as the preshift condition to OD_600_ ≈ 0.3 to 0.4. The cell cultures were then quickly collected by a 0.22 μm filter membrane using a vacuum filtration system (TW-606N, Toone Bio, Zhou Wen, China), further washed twice by 5 mL pre-warmed post-shift minimal medium. The filter membrane was then placed in a sterilized Petri dish containing 5 mL pre-warmed post-shift minimal medium. The cells were washed down by pipetting and quickly transferred into the post-shift medium as the transition time point at an initial OD_600_ ≈ 0.1. The entire transfer process generally took less than 3 min. The growth curve of postshift culture was then automatically monitored by a Synergy H1 microplater reader (Biotek). To quantify the lag time of nutrient downshift, the exponential range of the growth curve for the culture in postshift medium was analyzed by exponential fitting to get the exponential growth function (OD_600_ versus time), F_(postshift)_. The initial OD_600_ of postshift culture during transition point was designed as OD_ini_ (immediately measured after transition). Then with F_(postshift)_ and OD_ini_, we could obtain the time point at which the cells had just completely resumed the exponential growth, T_resume_. The lag time, T_lag_ = T_resume_-T_transition_, where T_transition_ is the transition point of postshift (the time when cells were just transferred into the postshift medium).

For this study, there were three types of nutrient downshift experiments in total. Two nitrogen (N) downshift experiments included glucose+NH_4_Cl medium shifted to glucose + alanine medium (NH_4_Cl to alanine), glucose+NH4Cl medium shifted to glucose + arginine medium (NH_4_Cl to arginine). One carbon (C) downshift was the shift from glucose+NH_4_Cl medium to alanine+NH_4_Cl medium.

#### Determination of the cellular ppGpp pools

The determination of the cellular ppGpp pools was based on HPLC methods as described in Zhu & Dai, Ryals et al.^6^^,^^66^ with modifications. *E. coli* cells were first exponentially growing to OD_600_ ≈ 0.4. 45 mL cultures were then fixed by 5 mL pre-chilled 1.8% formaldehyde on ice for 20–30 min. Cell pellets were then collected by centrifuge and hydrolyzed by 0.5 mL 0.6 M KOH for 30 min on ice. KOH was then neutralized by 15 μL of 85% phosphoric acid. The hydrolyzed samples were further centrifuged and the supernatants were filtered through a 0.45 μm Corning HPLC membrane filter (Cat No: 8161 or 8162). ppGpp samples (20–40 μL) were analyzed by Agilent 1260 HPLC machine with a C18 column (Polaris, C18-A, 180 Å, 5 μm, 4.6 × 250 mm) at a flow rate of 1 mL/min and monitored at 254 nm under 25°C. The gradient procedure was as below: 0 to 28 min: the fraction of acetonitrile (Buffer B) increased linearly from 18% to 30%. Mobile phase: buffer A: 0.03 M KH_2_PO_4_, 15 mM tetrabutylammonium phosphate, pH 6.5. Buffer B: 100% acetonitrile. The absolute quantification of ppGpp level was obtained with reference to the ppGpp standard samples (Tri-link).

#### Proteomics

Proteomics study followed exactly the same protocol as described in Zhu & Dai^6^ and described below again. 30 mL exponential culture of *E. coli* (OD_600_ ≈ 0.3) was transferred into a pre-cooled 50 mL centrifuge tube (Nest Biotech), and collected by centrifuge (4°C, 8500 rpm for 5 min). The cell pellets were washed twice by PBS, dried by a speed vacuum concentrator (CV600, Beijing JM Technology Co., Ltd.), and stored at −80°C freezer prior to proteomic analysis. The proteomics was based on 4D label-free mass spectrometry approach,^67^ which was performed by Jingjie PTM Biolabs (Hang Zhou). The experimental procedure of 4D label-free method was described as below: the cell pellets were subject to ultra-sonication in lysis buffer (8 M Urea 8 M urea, 1% Triton X-100, 10 mM DTT, 1% protease inhibitor cocktail and 2 mM EDTA). The cell debris was then removed by centrifuge (4°C, 12000 g) for 10 min. The supernatant was transferred into a new centrifuge tube for protein concentration measurement using BCA kit. Protein samples were next subject to trypsin digestion. For digestion, protein samples were first pelleted by 20% TCA at 4°C for 2 h and then collected with centrifuge (4500 g) for 5 min. The precipitates were washed for twice using pre-cooled acetone and further added with 200 mM TEAB. Trypsin was then added at 1:50 trypsin-to-protein mass ratio for digestion overnight. The solution was reduced with 5 mM DTT for 30 min at 56°C and alkylated with 11 mM iodoacetamide for 15 min at room temperature in darkness. Finally, the peptides were desalted by C18 SPE column. Solvent A (0.1% formic acid, 2% acetonitrile) and Solvent B (0.1% formic acid, 100% acetonitrile) were used for following peptides separation and UPLC procedures. The peptides were dissolved in solvent A and separated by the NanoElute UPLC system. The flow setting of UPLC was as follows: 0–43 min, 6%–22%B; 43–55 min, 22%–30%B; 55–58 min, 30%–80%B; 58–61 min, 80%B; flow rate: 450 nL/min. After the separation by UPLC, the peptide was set into Capillary ionization source for ionization and further analyzed by timsTOF Pro mass spectrometry system. The electrospray voltage applied was set at 1.6 to 1.8 kV. Both the original peptide ion and its secondary fragments were detected and analyzed by high-resolution TOF. The m/z scan range was 100–1700 for full scan. Precursors with charge states 0 to 5 were selected for fragmentation, and 10 PASEF-MS/MS scans were acquired per cycle. The dynamic exclusion was set to 30 s. The mass spectra data were searched against the SwissProt *E. coli* K-12 databases and analyzed by Maxquant software,^68^ which gave the information of both LFQ intensity and iBAQ intensity. The information of the relative abundance of each protein across different conditions was given by LFQ intensity. The mass proteome fraction (absolute abundance) of individual proteins was obtained using the iBAQ intensity of each protein to multiply the molecular weight (MW) (we referred to as “iBAQ mass” in supplementary table) and further normalized by the sum of the whole proteome (as iBAQ intensity is a proxy of the copy number of each protein, therefore, iBAQ time MW could reflect the mass of each protein).^42^^,^^69^ The iBAQ mass of individual proteins, together with the gene locus-tag were submitted to proteomaps website to obtain the KEGG resource allocation map of *E. coli* cells.^44^

### Quantification and statistical analysis

The raw data of mass spectra was analyzed and quantified using the by Maxquant v1.6.15.0 software to give both LFQ intensity and iBAQ intensity. Statistical analysis was carried out using GraphPad Prism software version 9.0.0.

## Supplemental information

Document S1. Figures S1–S3

Table S1. A systematic annotation of proteomic data of RE198LQ provided by the Jingjie PTM Biolabs, related to Figures 1, 2, and 4 The relative abundances of each protein (column AD to AG) correspond to exactly the data of LFQ intensity (column Z to AC). It is obtained by using the LFQ intensity of each condition to be normalized by the average of the LFQ intensity of all conditions of each protein.

Table S2. Absolute abundance of each individual protein in REL198LQ project with the information of iBAQ intensity, related to Figures 1, 2, and 4 Maxquant raw data gives the information of iBAQ intensity, which is a proxy of the copy number of each protein. We then use the iBAQ intensity of each protein to multiply its molecular weight (MW) to obtain the iBAQ mass. The proteome fraction of each protein (or a protein function sector) is obtained by normalizing its value by the sum of the whole proteome.

Table S3. The proteome fraction of each proteome function sector in RE198LQ project using the information of iBAQ mass in Table S2, related to Figures 1, 2, and 4

Table S4. The proteome fraction of various stress-responsive function sectors in RE198LQ project using the information of iBAQ mass in Table S2, related to Figures 1, 2 and 4 For the groups of [GO:0006950 (Response to stress)] and [GO:0051716 (Cellular response to stimulus)], the gene lists were obtained by submitted the ID of those upregulated proteins (upregulated by 1.5-fold based on the LFQ intensity) to (http://geneontology.org) for Gene ontology enrichment analysis.

Table S5. The proteome fraction of rpoS-regulon genes in RE198LQ project using the information of iBAQ mass in Table S2, related to Figures 1, 2, and 4 Two versions of gene list were used based on [Patten et al 2004 Mol Genet Genomics 272(5):580-591] and [Weber et al 2005. J Bacteriol 187(5):1591-1603].

Table S6. A systematic annotation of proteomic data of XA00994LQ project provided by the Jingjie PTM Biolabs, related to Figure 4 The relative abundances of each protein (column AN to AS) correspond to exactly the data of LFQ intensity (column AH to AM). It is obtained by using the LFQ intensity of each condition to be normalized by the average of the LFQ intensity of all conditions of each protein.

Table S7. The absolute abundance of each individual protein in XA00994LQ project with the information of iBAQ intensity, related to Figure 4 Maxquant raw data gives the information of iBAQ intensity, which is a proxy of the copy number of each protein. We then use the iBAQ intensity of each protein to multiply its molecular weight (MW) to obtain the iBAQ mass. The proteome fraction of each protein (or a protein function sector) is obtained by normalizing its value by the sum of the whole proteome.

Table S8. The proteome fraction of each proteome function sector in XA00994LQ project using the information of iBAQ mass in Table S7, related to Figure 4

Table S9. The proteome fraction of various stress-responsive function sectors in XA00994LQ project using the information of iBAQ mass in Table S7, related to Figure 4 The gene list of [GO:0006950 (Response to stress)] and [GO:0051716 (Cellular response to stimulus)] are the same according to Table S4.

Table S10. The proteome fraction of rpoS-regulon genes in XA00994LQ project using the information of iBAQ mass in Table S7, related to Figure 4 Two versions of gene list were used based on [Patten et al 2004 Mol Genet Genomics 272(5):580-591] and [Weber et al 2005. J Bacteriol 187(5):1591-1603].
