# Supplementary material for: Integrated control of bacterial growth and stress response by (p)ppGpp in Escherichia coli: A seesaw fashion
Source: iScience. 2024 Jan 9;27(2):108818. doi: 10.1016/j.isci.2024.108818 (PMC10828813; doi:10.1016/j.isci.2024.108818)
Supplement: Document S1. Figures S1–S3 [file mmc1.pdf]

iScience, Volume *27*

Supplemental information

Integrated control of bacterial growth and stress response by (p)ppGpp in *Escherichia coli*: A seesaw fashion

Manlu Zhu, Haoyan Mu, and Xiongfeng Dai

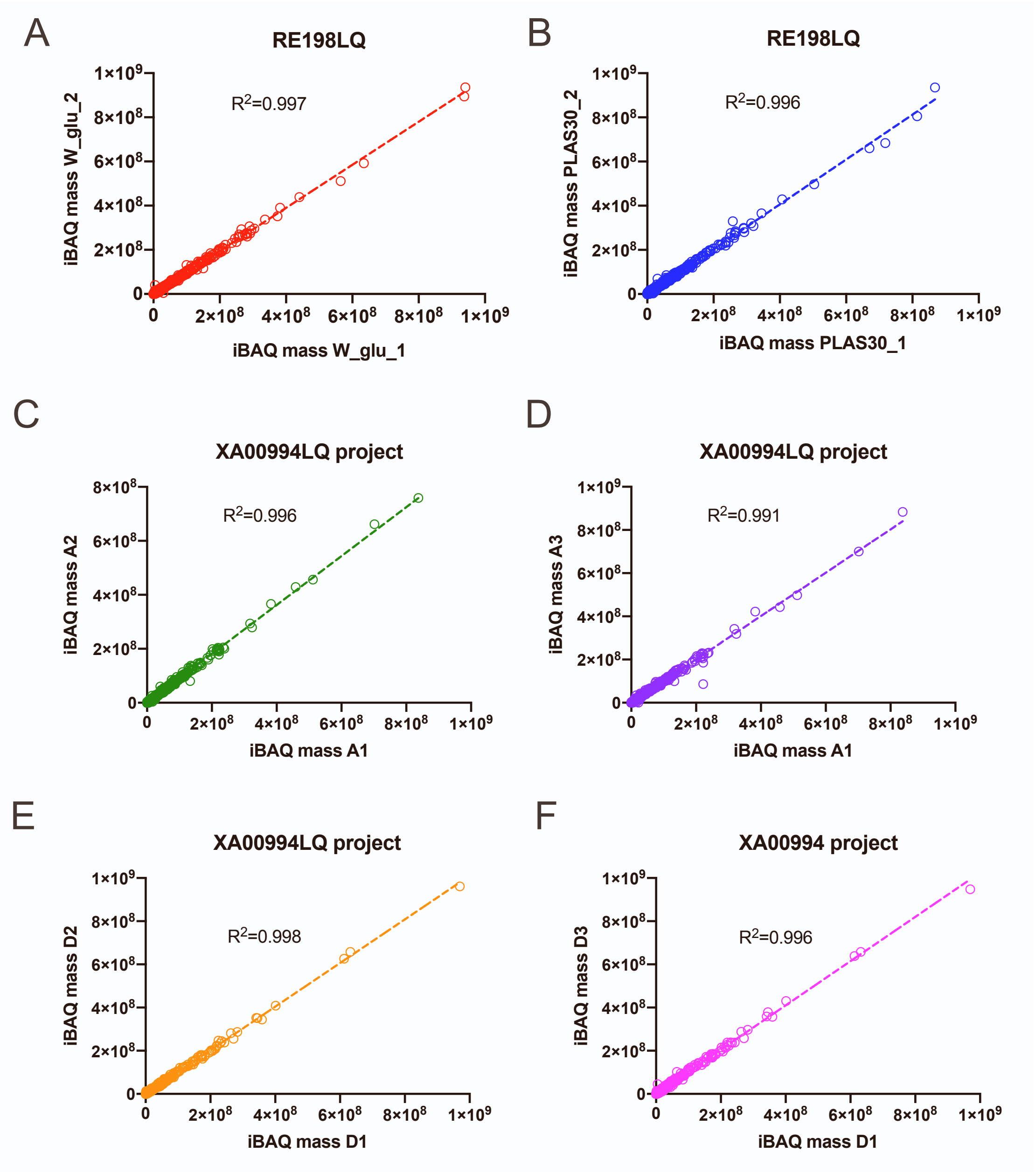

Figure S1. Reproducibility of the proteome raw data measured by 4D label-free mass spectrometry. Related to Figure 2 and Figure 4. The conditions of wild type *E. coli* strain (pane A) and its RelA* OE strain (panel B) growing in glucose minimal medium had both been done for twice (samples of two biological replicates), see RE198LQ project of supplementary table for details. The conditions of *rpoS*-null *E. coli* strain (pane C and D) and its RelA* OE strain (panel E and F) growing in glucose minimal medium had both been done for three times (samples of three biological replicates), see XA00994LQ project of supplementary table for details. We analyzed the relative proteome mass of each individual proteins using the information of iBAQ intensity×MW (molecular weight) (see method and supplementary table). We then plotted different replicates against with each other and confirmed the high reproducibility of different replicates.

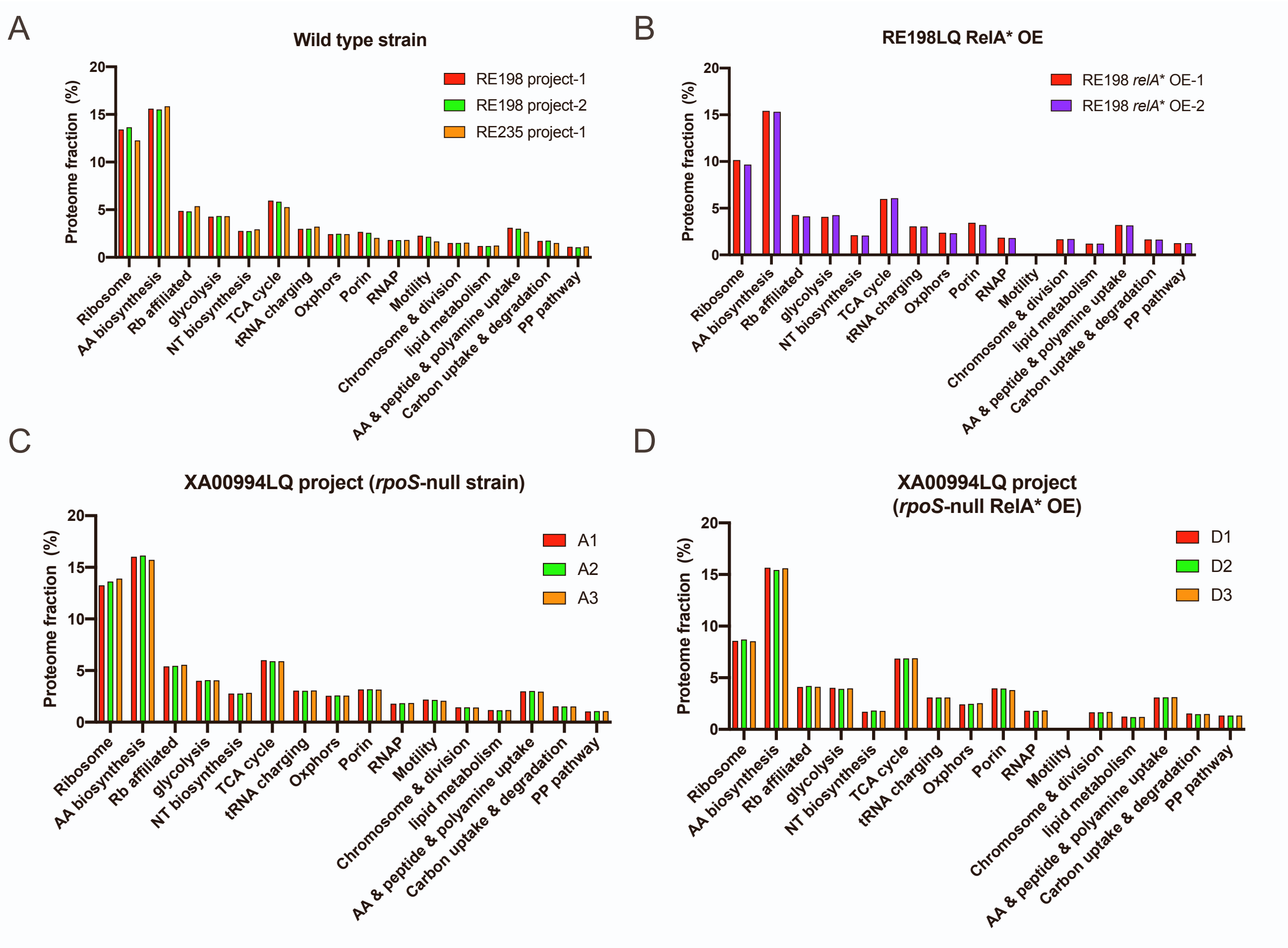

Figure S2. Reproducibility of the proteome fractions of major proteome functional sectors. Related to Figure 2 and Figure 4. Conditions are the same as described above in supplementary Figure 1, and see details in supplementary data 3 and supplementary data 8. In addition, the raw data of RE235 project could be find in the supplementary table of Zhu & Dai (2023) Nat Commun 14, 467. From the data, we can find that all the replicates show very similar results with each other with the standard deviation being within 10%.

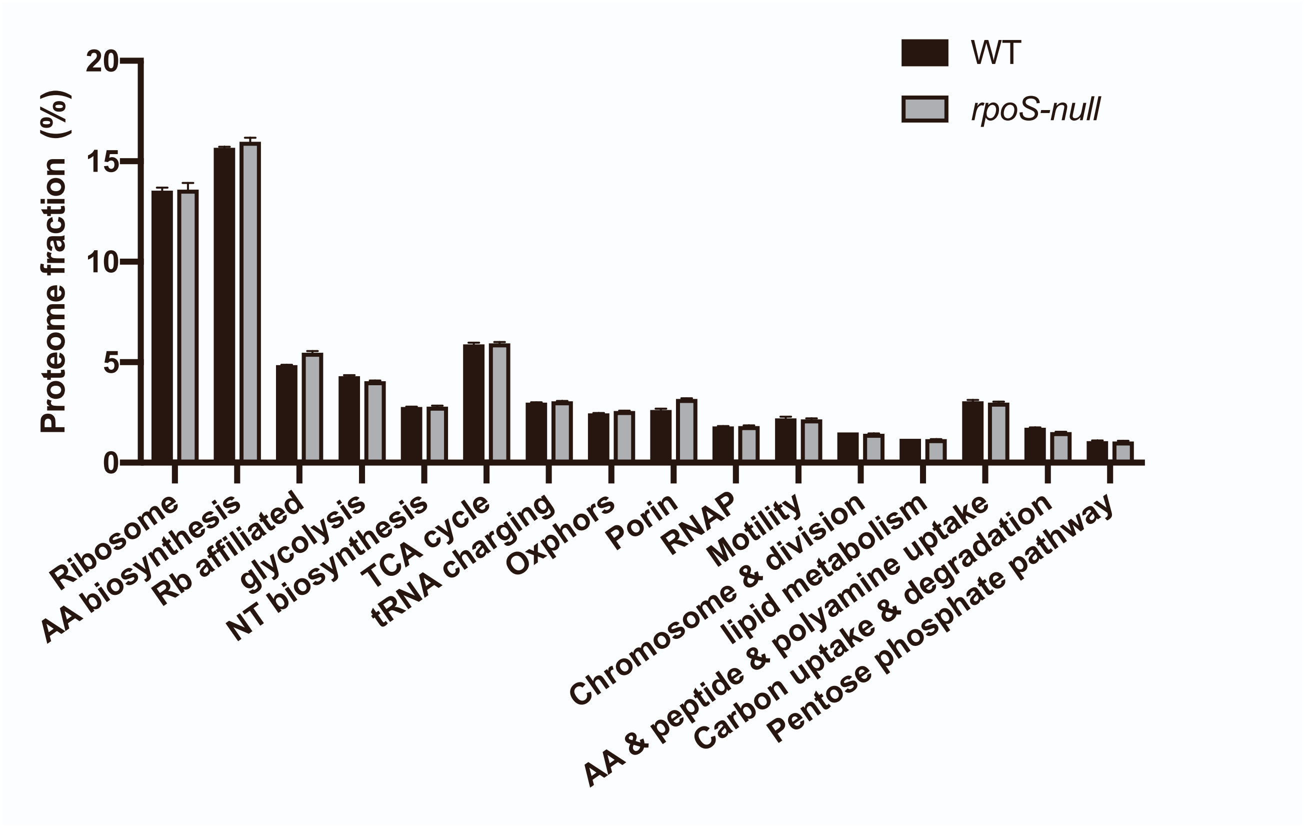

Figure S3. The mass fractions of various proteome sectors in wild type strain and *rpoS*-null strain.

Related to Figure 4.
